# One symptom, two arrhythmias: the rare and the even rarer

**DOI:** 10.1186/s12872-017-0679-y

**Published:** 2017-09-12

**Authors:** Florian Zauner, Jan-Thorben Sieweke, Stephan Hohmann, David Duncker, Christian Riehle, L. Christian Napp, Ulrike Flierl, Thorben König, Christian Veltmann

**Affiliations:** Department of Cardiology & Angiology, Hannover Medical School, Carl-Neuberg-Strasse 1, D-30625 Hannover, Germany

## Abstract

**Background:**

Wolff-Parkinson-White (WPW) syndrome and idiopathic left ventricular tachycardia (ILVT) are rare and up to now the coexistence of both entities has rarely been reported.

In patients with ventricular preexcitation the underlying mechanism of paroxysmal tachycardia most likely is atrioventricular reentrant tachycardia (AVRT). However, without ECG documentation of the tachycardia diagnosis of the underlying mechanism cannot be made due to similar clinical presentation of AVRT and ILVT.

**Case presentation:**

We report a case of a two-staged occurrence of two rare arrhythmias in a young adult, who was admitted to our hospital twice within 6 months because of paroxysmal tachycardia. WPW syndrome and ILVT as underlying arrhythmias have been diagnosed and were ablated successfully.

**Conclusions:**

This case highlights the diagnostic defiance of rare tachycardia entities and the paramount importance of ECG documentation and analysis of all available tachycardia ECGs.

## Background

Paroxysmal tachycardias with regular heartbeat in the young without structural heart disease are most commonly caused by atrioventricular-nodal reentrant tachycardia (AVNRT) and atrioventricular reentrant tachycardia (AVRT). Wolff-Parkinson-White (WPW) syndrome and idiopathic left ventricular tachycardia (ILVT) have a similar clinical presentation. This case highlights the diagnostic defiance of rare tachycardia entities especially in the preclinical setting.

## Case presentation

**Fig. 1:**
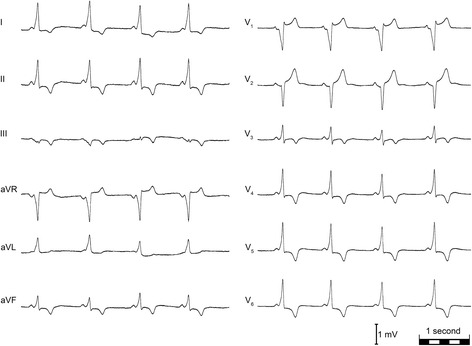
12-lead ECG shows sinus rhythm, normal axis, 60 bpm, shortened PQ (112 ms), broadened QRS (122 ms), prominent delta waves in I, II, aVL, aVF, V_4_-V_6_

A 41-year old male patient with an oligosymptomatic wide complex tachycardia was referred to the emergency department. The tachycardia was terminated by a 360 J DC-shock after unsuccessful administration of adenosine prehospitally. ECG documentation of the tachycardia was not available. The patient had no history of relevant comorbidities. Physical exam was unremarkable, the patient did not take any medication. Similar episodes with palpitations over several minutes and one syncope in the past were reported. Initial 12-lead ECG showed a shortened PR interval with prominent delta waves in I, II, aVL, V_4_-V_6_ and R/S transition at V_4_/V_5_ suggesting a right midseptal pathway (Fig. 1). Diagnosis of WPW syndrome was established. Non-invasive cardiologic work-up did not show significant pathologies.

AVRT was suspected as the underlying mechanism of arrhythmia. Due to recurrent symptomatic paroxysmal tachycardias an electrophysiological study (EPS) was scheduled. An accessory pathway with bidirectional conduction in right-midseptal location was diagnosed and successfully ablated. After ablation no tachycardia could be induced by atrial and ventricular stimulation at rest and following isoprenaline infusion. The patient was discharged the next day.

**Fig. 2:**
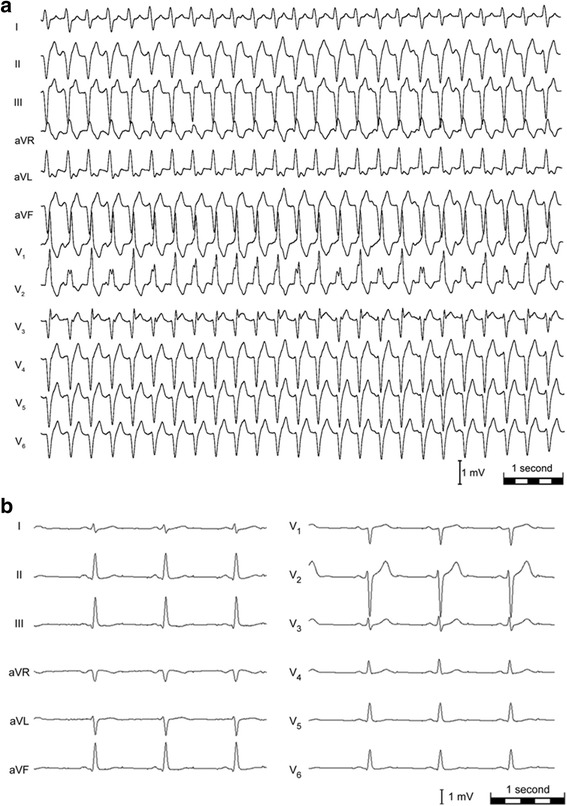
**a** 12-lead ECG shows ILVT with right bundle branch block morphology (QRS 130 ms), left axis deviation, 168 bpm. **b** 12-lead ECG after ablation of the accessory pathway and the ILVT

Five months later the patient was admitted to the emergency department complaining about palpitations and dyspnea since the evening before. ECG showed a wide complex tachycardia (168 beats per minute (bpm); QRS duration 130 ms) with right bundle branch block (RBBB) morphology, left axis deviation and AV (atrioventricular) dissociation (Fig. 2a). Diagnosis of an ILVT was made. Following administration of Verapamil (cumulative: 240 mg p.o. and 40 mg i.v.) tachycardia slowed down and converted into sinus rhythm. After hospital admission the patient remained in sinus rhythm on Verapamil (120 mg sustained release b.i.d.) and was discharged 1 day later. EPS and ablation of ILVT was recommended. At scheduled ablation the patient presented the ECG of the index-tachycardia, which was terminated by DC-shock. This ECG showed the ILVT rather than the initially suspected AVRT.

In the EPS ILVT of the common type was induced by atrial burst-stimulation and radiofrequency ablation of the ILVT was performed. The patient showed normal AV conduction without signs of pre-excitation. Figure 2b displayed ECG after ablation of WPW syndrome and ILVT. There were no palpitations or symptoms during a follow up of 18 months.

## Discussion and conclusions

Paroxysmal tachycardia is a common cause for emergency admission in adults. In young patients with a structurally normal heart, the most common cause is AVNRT, followed by AVRT. Especially in the presence of a delta wave in the 12-lead ECG, AVRT is most likely the mechanism of the paroxysmal tachycardias [1, 2]. However, determining the correct underlying rhythm might be challenging.

The present case offers an exceptional constellation, providing the consecutive occurrence of two rare arrhythmias with identical clinical presentation in a short time interval. Due to the history of tachycardia and a pathognomonic ECG for WPW syndrome we initially expected an AVRT as underlying mechanism.

At second admission ILVT was found, which is also predominantly found in young males without structural heart disease. The mechanism of this tachycardia is related to the Purkinje network with a macroreentry involving the anterior, posterior or septal fascicle of the left ventricle [3]. ILVT is an even more infrequent arrhythmia, which might be misdiagnosed as SVT with left axis deviation and RBBB, especially in case of 1:1 retrograde conduction to the atria.

Adenosine was applied without any effect. The origin of wide complex tachycardias can be ventricular or supraventricular. As shown in the present case, in hemodynamically stable patients, Adenosine can be used in regular wide complex tachycardias, to diagnose the mechanism or terminate the tachycardia [2].

Catheter ablation represents an efficient and safe therapeutic option for ILVT as well as in WPW syndrome [1, 2].

In summary, this case illustrates the defiance of differential diagnosis in patients without structural heart disease presenting with paroxysmal tachycardias with wide QRS complex. Diagnosis of ILVT is impossible without ECG documentation. Wide complex tachycardias with RBBB, left anterior fascicular block and a narrow/moderately widened QRS complex are suspicious for ILVT. This case shows the paramount importance of ECG documentation and analysis of all available tachycardia ECGs.

## Abbrevations

AV: Atrioventricular
AVNRT: AV-nodal-reentrant tachycardia
AVRT: AV reentrant tachycardia
Bpm: Beats per minute
EPS: Electrophysiological study
ILVT: Idiopathic left ventricular tachycardia
RBBB: Right bundle branch block
SVT: Supraventricular tachycardia
VT: Ventricular tachycardia
WPW: Wolff-Parkinson-White
